# Supplementary material for: Epidemiology and Integrative Taxonomy of Helminths of Invasive Wild Boars, Brazil
Source: Pathogens. 2023 Jan 23;12(2):175. doi: 10.3390/pathogens12020175 (PMC9963619; doi:10.3390/pathogens12020175)
Supplement: Supplementary file 1 [file pathogens-12-00175-s001.zip › Table S1.pdf]

Table S1. Descriptors of helminth infection in wild boars (*Sus scrofa*) from São Paulo state, Brazil.

Helminths	Site of infection	Prevalence (%)	Mean Abundance ± SD	Mean intensity ± SD	Range of Intensity
**NEMATODA**					
**Ancylostomatoidea**					
Globocephalus urosubulatus	Small intestine	94.3	215.5 ± 31.7	228.6 ± 32.2	1 - 892
**Strongyloidea**					
Stephanurus dentatus	Peritoneum, ureters, kidneys, liver, heart	71.4	17.9 ± 3.8	25.1 ± 4.6	1 - 341
Oesophagostomum dentatum	Large intestine	2.9	0.03	1	-
**Metastrongyloidea**					
Metastrongylus salmi	Lungs	82.9	25.9 ± 5.8	31.2 ± 6.6	1 - 128
Metastrongylus pudendotecus	Lungs	11.4	0.26 ± 0.1	2.25 ± 0.6	1 - 4
**Rhabditoidea**					
Strongyloides ransomi	Small intestine	59.3	118.3 ± 32.6	188.2 ± 46	1 - 673
**Spiruroidea**					
Ascarops strongylina	Stomach	28.6	0.57 ± 0.2	2 ± 0.6	1 - 6
**Trichuroidea**					
Trichuris suis	Large intestine	8.6	0.23 ± 0.1	2.7 ± 0.7	1 - 8
**Ascaridoidea**					
Ascaris suum	Small intestine	2.9	0.03	1	-
**ACANTOCEPHALA**					
**Oligacanthorrhychidae**					
Macracanthorhynchus hirudinaceus	Small intestine	5.7	0.2±0.2	3.5±2.5	1 – 6

SD: standard deviation
