# Supplementary material for: Epidemiology and Integrative Taxonomy of Helminths of Invasive Wild Boars, Brazil
Source: Pathogens. 2023 Jan 23;12(2):175. doi: 10.3390/pathogens12020175 (PMC9963619; doi:10.3390/pathogens12020175)
Supplement: Supplementary file 1 [file pathogens-12-00175-s001.zip › Table S2.pdf]

Table S2: Morphometric data of *Ascarops strongylina* by different authors, presented as mean ± standard deviation, in millimeters

	**This study (n=10)**	**Dakova and Panayotova-Pencheva [19]**
**Host**	Wild boar	Pig
**Male**		
Length	13.4 ± 0.73	12
Width	0.336 ± 0.005	0.33
Pharynx	0.10 ± 0.01	0.086
Esophagus	3.65 ± 0.12	3.2
Excretory pore	0.295 ± 0.023	-
Nerve ring	0.256 ± 0.012	-
Smaller spicule	0.515 ± 0.117	0.575
Bigger spicule	2.940 ± 0.151	2.1
Cloaca to the posterior end	0.25 ± 0.037	0.253
Gubernacle length	0.071 ± 0.08	0.055
Gubernacle width	0.054 ± 0.01	0.038
Caudal ala width	0.33 ± 0.093	0.29
**Female**		
Length	17.945 ± 1.09	16.52 ± 2.76
Width	0.375 ± 0.033	0.357.8 ± 0.037
Pharynx	0.11 ±0.008	0.083 ± 0.008
Esophagus	3.460 ± 0.48	3.36 ± 0.4
Excretory pore	0.417 ± 0.011	-
Nerve ring	0.288 ± 0.03	-
Vulva to the anterior end	8.525 ± 0.6	8.120 ± 1.34
Anus to the posterior end	0.305 ± 0.017	0.232 ± 0.059
