# Supplementary material for: Epidemiology and Integrative Taxonomy of Helminths of Invasive Wild Boars, Brazil
Source: Pathogens. 2023 Jan 23;12(2):175. doi: 10.3390/pathogens12020175 (PMC9963619; doi:10.3390/pathogens12020175)
Supplement: Supplementary file 1 [file pathogens-12-00175-s001.zip › Table S3.pdf]

Table S3: Morphometric data of parthenogenetic females of *Strongyloides ransomi* by different authors, presented as mean ± standard deviation, in millimeters

	**This study (n=10)**	**Alicata [20]**	**Giang et al. [21]**
**Host**	Wild boar	Pig	Pig
**Female**			
Length	5.26 ± 0.276	3.3 – 4.5	4.5 – 5.0
Width	0.09 ± 0.008	0.054 – 0.062	0.052 – 0.060
Nerve ring	0.25 ± 0.015	–	–
Esophagus	1.015 ± 0.026	0.61 – 0.88	0.940 – 1.1
Vulva to the anterior end	3.26 ± 0.159	1.9 – 2.9	2.6 – 3.1
Anus to the posterior end	0.08 ± 0.005	0.053 – 0.083	0.055 – 0.075
