# Supplementary material for: Epidemiology and Integrative Taxonomy of Helminths of Invasive Wild Boars, Brazil
Source: Pathogens. 2023 Jan 23;12(2):175. doi: 10.3390/pathogens12020175 (PMC9963619; doi:10.3390/pathogens12020175)
Supplement: Supplementary file 1 [file pathogens-12-00175-s001.zip › Table S4.pdf]

Table S4: Morphometric data of *Globocephalus urosubulatus* by different authors, presented as mean ± standard deviation, in millimeters

	**This study (n=10)**	**Francis [22]**	**Nanev et al. [23]**	**Pinheiro et al. [24]**
**Host**	Wild boar	Pig	Wild boar	Pig
**Male**				
Length	6.17 ± 0.471	3.16-5.32	3.5-5	4 – 5
Width	0.35 ± 0.023	0.320 – 0.400	0.360 – 0.370	0.167 – 0.300
Buccal capsule length	0.15 ± 0.014	0.119 – 0.127	0.140 – 0.200	0.125 – 0.150
Buccal capsule width	0.13 ± 0.023	0.162 – 0.186	0.150 – 0.170	0.100 – 0.140
Excretory pore	0.49 ± 0.04	0.320 – 0.500	–	0.317 – 0.417
Nerve ring	048 ± 0.039	0.290 – 0.460	0.380 – 0.520	0.317 – 0.367
Cervical papillae	0.56 ± 0.042	–	0.430 – 0.610	0.370 – 0.533
Esophagus	0.62 ± 0.026	0.550 – 0.710	0.560 –0.690	0.487 – 0.540
Espicules	0.61 ± 0.09	0.490 – 0.550	0.420 – 0.580	0.337 – 0.527
Gubernacle	0.08 ± 0.001	0.089 – 0.110	0.070 – 0.080	0.060 – 0.088
**Female**				
Length	8.4 ± 0.27	5.02-6.53	4.5-8	6-8
Width	0.47 ± 0.054	0.490 – 0.570	0.420 – 0.500	0.429 – 0.514
Buccal capsule length	0.21 ± 0.017	0.192 – 0.220	0.140 – 0.200	0.167 – 0.227
Buccal capsule width	0.14 ± 0.02	0.159 – 0.178	0.150 – 0.170	0.140 – 0.160
Excretory pore	0.65 ± 0.02	0.320 – 0.530	–	0. 433 – 0.547
Nerve ring	0.63 ± 0.035	0.380 – 0.460	0.380 – 0.520	0.387 – 0.500
Cervical papillae	0.71 ± 0.017	–	0.430 – 0.610	0.433 – 0.547
Esophagus	0.84 ± 0.11	0.680 – 0.75	0.560 –0.690	0.593 –0.687
Vulva to the posterior end	6.67± 0.023	1.73-2.91	2.20-2.40	3-5
Anus to the posterior end	0.22 ± 0.034	–	0.120 – 0.180	0.130 – 0.200
