# Supplementary material for: Epidemiology and Integrative Taxonomy of Helminths of Invasive Wild Boars, Brazil
Source: Pathogens. 2023 Jan 23;12(2):175. doi: 10.3390/pathogens12020175 (PMC9963619; doi:10.3390/pathogens12020175)
Supplement: Supplementary file 1 [file pathogens-12-00175-s001.zip › Table S5.pdf]

**Table S5:** Morphometric data of *Oesophagostomum dentatum* by different authors, presented as mean ± standard deviation, in millimeters

| | **This study (n=10)** | **Dakova e Panayotova-Pencheva [19]** |
|---|---|---|
| **Host** | Wild boar | Wild boar |
| **Male** | | |
| Length | 8.98 ± 0.038 | 9.46 ± 0.28 |
| Width | 0.21 ± 0.018 | 0.218 ± 0.014 |
| Excretory pore | 0.34 ± 0.023 | – |
| Nerve ring | 0.24 ± 0.008 | – |
| Esophagus length | 0.43 ± 0.017 | 0.397 ± 0.104 |
| Esophagus width | 0.13 ± 0.013 | 0.110 ± 0.007 |
| Spicules | 0.98 ± 0.118 | 1.135 ± 0.030 |
| Gubernacle | 0.10 ± 0.007 | 0.127 ± 0.006 |
| **Female** | | |
| Length | 12.88 ± 0.409 | 11.5 ± 0.6 |
| Width | 0.24 ± 0.008 | 0.233 ± 0.014 |
| Excretory pore | 0.345 ± 0.021 | – |
| Nerve ring | 0.24 ± 0.023 | – |
| Esophagus length | 0.45 ± 0.02 | 0.426 ± 0.102 |
| Esophagus width | 0.12 ± 0.008 | 0.115 ± 0.005 |
| Vulva to the posterior end | 0.63 ± 0.113 | – |
| Vulva to the anus | 0.29 ± 0.021 | 0.347 ± 0.015 |
| Anus to the posterior end | 0.27 ± 0.043 | 0.293 ± 0.007 |
