# Supplementary material for: Epidemiology and Integrative Taxonomy of Helminths of Invasive Wild Boars, Brazil
Source: Pathogens. 2023 Jan 23;12(2):175. doi: 10.3390/pathogens12020175 (PMC9963619; doi:10.3390/pathogens12020175)
Supplement: Supplementary file 1 [file pathogens-12-00175-s001.zip › Table S6.pdf]

Table S6. Morphometric data of *Trichuris suis* by different authors, presented as mean ± standard deviation, in millimeters

	**This study (n=10)**	**Cutillas et al. [25]**		**Nissen et al. [26]**
**Host**	Wild boar	Pig	Wild boar	Pig
**Male**				
Length	36.784 ± 3.854	40.4 ± 4.2	40.8 ± 8.8	28.9
Esophagus	2.346 ± 0.29	2.61 ± 0.31	2.5 ± 0.58	-
Width of the anterior end	0.184 ± 0.07	0.20 ± 0.02	0.20 ± 0.03	0.16
Width at the esophagus-intestine junction	0.379 ± 0.08	0.35 ± 0.04	0.38 ± 0.06	-
Width of the posterior end	0.725 ± 0,098	0.87 ± 0.06	0.83 ± 0.13	0.58
Spicule	2.37 ± 0.182	2.35 ± 0.15	2.43 ±0.22	2.21
Spicule sheath	0.29 ± 0.132	0.34 ± 0.20	0.15 ± 0.09	-
**Female**				
Length	44.77 ± 3.8	44.3 ± 2.8	46.3 ± 5.3	28.5
Esophagus	3.268 ± 0.201	3.12 ± 0.16	3.28 ± 0.39	-
Width of the anterior end	0.21 ± 0.04	0.19 ± 0.01	0.20 ± 0.02	0.18
Width at the esophagus-intestine junction	0.34 ± 0.08	0.36 ± 0.03	0.30 ± 0.08	-
Width of the posterior end	0.73 ± 0.11	0.93 ± 0.10	0.90 ± 0.08	0.69
Vulva to the esophagus-intestine junction	0.25 ± 0.05	0.23 ± 0.08	0.25 ± 0.022	-
