# Supplementary material for: Epidemiology and Integrative Taxonomy of Helminths of Invasive Wild Boars, Brazil
Source: Pathogens. 2023 Jan 23;12(2):175. doi: 10.3390/pathogens12020175 (PMC9963619; doi:10.3390/pathogens12020175)
Supplement: Supplementary file 1 [file pathogens-12-00175-s001.zip › Table S7.pdf]

Table S7: Morphometric data of *Macracanthorhynchus hirudinaceus* by different authors, presented as maximum and minimum values, in millimeters

	This study (n=3)	Lisitsyna [27]	Amin et al. [28]
**Host**	Wild boar	Wild boar	Wild boar
**Female**			
Length	304 – 381	105 – 500	110 – 120
Width	6 - 9	7 - 9	5 – 6
Number of hook rows	6	6	5 - 6
Number of hooks per row	6	5 - 6	5 - 6
