# Supplementary material for: Epidemiology and Integrative Taxonomy of Helminths of Invasive Wild Boars, Brazil
Source: Pathogens. 2023 Jan 23;12(2):175. doi: 10.3390/pathogens12020175 (PMC9963619; doi:10.3390/pathogens12020175)
Supplement: Supplementary file 1 [file pathogens-12-00175-s001.zip › Table S8.pdf]

Table S8. Primers used in the PCR reactions

Região	Nome	T°C	pb	Sequência	Referência
**Nematoda**					
18S1 rRNA	**988**-F	50°C	1500	5′- CTCAAAGATTAAGCCATGC-3′	Holterman et al., 2006
	**1912**-R			5′- TTTACGGTCAGAACTAGGG-3′	
18S2 rRNA	**1813**-F	52°C	1500	5′-CTGCGTGAGAGGTGA AAT-3′	
	**2646**-R			5′-GCTACCTTGTTACGACTTTT-3′	
ITS region	**NC5**-F	54,5°C	800	5′-GTAGGTGAACCTGCGGAAGGATCATT-3′	Gasser et al., 1993
	**NC2**-R			5′-TTAGTTTCTTTTCCTCCGCT-3′	
28S rRNA	**D2A-**F	56°C	770	5′-ACAAGTACCGTGAGGGAAAGTTG-3′	De Ley et al., 1999
	**D3B**-R			5′-TCGGAAGGAACCAGCTACTA-3′	

**Acantocephala**					
ITS region	**LSU-5-**F	56°C	1400	5′-TAGGTCGACCCGCTGAAYTTAAGCA-3′	Olson et al., 2003
	**1500R-**R			5′-GCTATCCTGAGGGAAACTTCG-3′	
28S rRNA	**BD1-**F	49°C	850	5′-GTCGTAACAAGGTTTCCGTA-3′	Luton et al., 1992
	**BD2-**R			5′ -TATGCTTAAATTCAGCGGGT-3′	
