# Supplementary material for: Epidemiology and Integrative Taxonomy of Helminths of Invasive Wild Boars, Brazil
Source: Pathogens. 2023 Jan 23;12(2):175. doi: 10.3390/pathogens12020175 (PMC9963619; doi:10.3390/pathogens12020175)
Supplement: Supplementary file 1 [file pathogens-12-00175-s001.zip › Table S9.pdf]

Table S9. Major genetic identities of 18S rDNA, 28S rDNA, and ITS regions sequences of helminths from the gastrointestinal and urinary tracts of wild boars hunted in the state of São Paulo, Brazil, with sequences deposited in the NCBI database.

Species	bp	Host, Location	Identy	E value	Query Cover	Acession number
**Nematoda**						
**18S rRNA**						
Ascarops strongylina	1773	*Streptopharagus* sp. (*Macaca mulata*, Guilin, China)	98,71%	0.0	100%	HM067977
Strongyloides ransomi	1539	*S. ransomi* (*Sus scrofa*, Wakayama, Japão)	99,94%	0.0	80%	AB453327
Globocephalus urosubulatus	1599	*Ancylostoma caninum* (*Canis familiaris*, não informado)	99%	0.0	99%	AJ920347
Oesophagostomum dentatum	753	*Oesophagostomum muntiacum* (*Muntiacus reevesi*, Tokyo, Japão)	100%	0.0	100%	LC415112
Trichuris suis	1728	*T. suis* (*Sus scrofa domestica*, Espanha)	100%	0.0	100%	HF586905
Stephanurus dentatus	1587	*S. dentatus* (Não informado)	100%	0.0	100%	MW979564
**ITS region**						
Globocephalus urosubulatus	790	*Ancylostoma caninum* (*Canis lupus familiaris*, EUA)	90,58%	0.0	100%	JQ812694
Oesophagostomum dentatum	726	*O. dentatum* (*Sus scrofa*, Yangjiang, China)	99,86%	0.0	94%	AJ619979
Stephanurus dentatus	580	*Strongylus edentatus* (*Equus asinus*, Henan, China)	83.42%	6e-151	78%	KP693438
**28S rRNA**						
Ascarops strongylina	991	*Cylicospirura petrowi* (*Felis silvestris*, Taunus, Alemanha)	89,29%	0.0	100%	KM434335
Globocephalus urosubulatus	605	*Ancylostoma caninum* (*Canis familiaris*, não informado)	98,84%	0.0	100%	AM039739
Oesophagostomum dentatum	599	*Oesophagostomum muntiacum* (*Muntiacus reevesi*, Tokyo, Japão)	99,00%	0.0	99%	LC415112
Stephanurus dentatus	621	*S. dentatus* (*Sus scrofa*, não informado)	100%	0.0	100%	MW979564
**Acantocephala**						
**28S rRNA**						
Macracanthorhynchus hirudinaceus	1032	*M. hirudinaceus* (*Sus scrofa leucomystax*, Yamaguchi, Japão)	98,55%	0.0	100%	LC350002
**ITS region**						
Macracanthorhynchus hirudinaceus	585	*M. hirudinaceus* (*Sus scrofa leucomystax*, Yamaguchi, Japão)	94,41%	0.0	100%	LC350000
