# Supplementary figures and images for: Epidemiology and Integrative Taxonomy of Helminths of Invasive Wild Boars, Brazil
Source: Pathogens. 2023 Jan 23;12(2):175. doi: 10.3390/pathogens12020175 (PMC9963619; doi:10.3390/pathogens12020175)

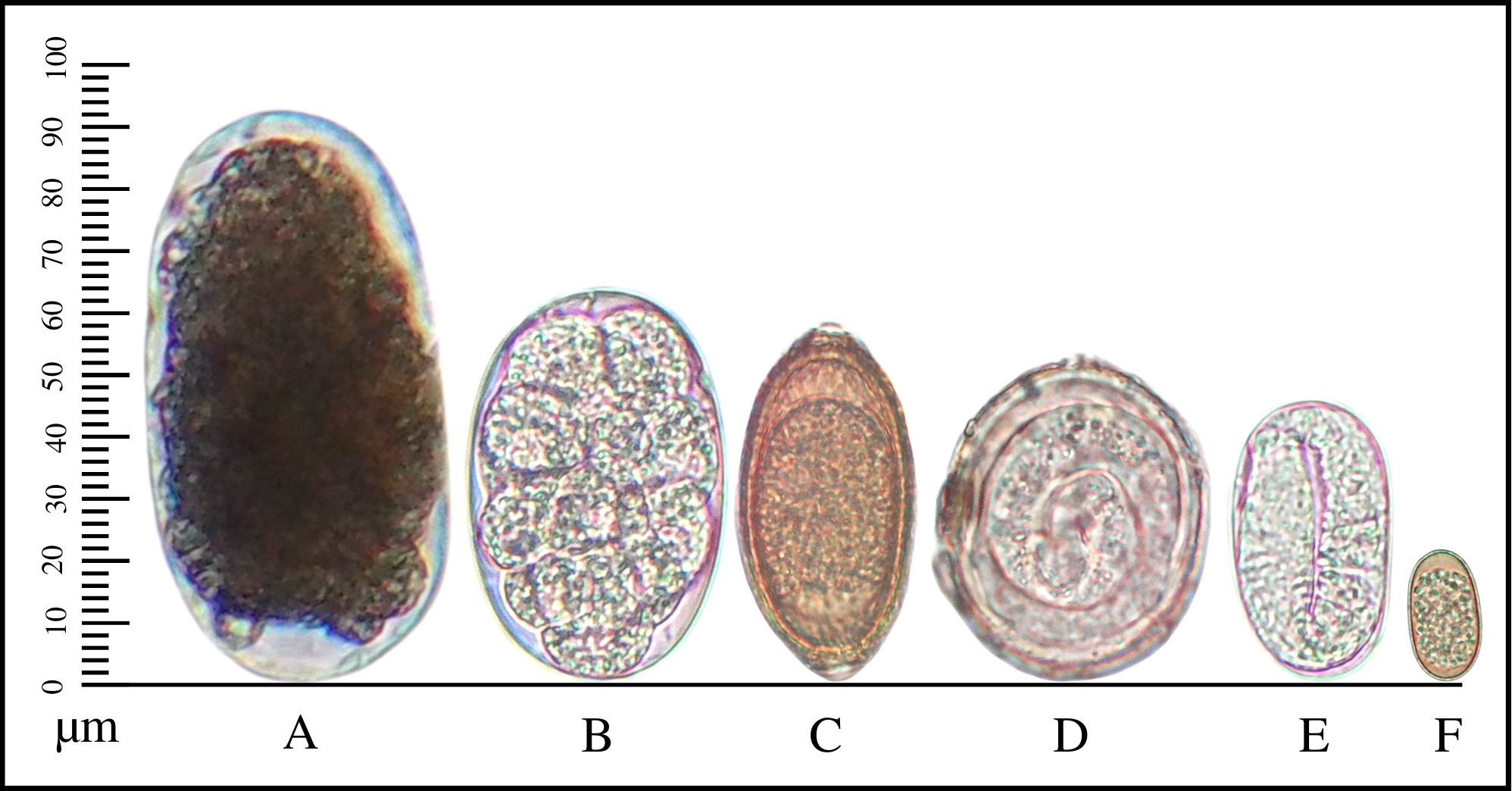

Supplement: Supplementary file 1 [file pathogens-12-00175-s001.zip › Figure S1.tiff]

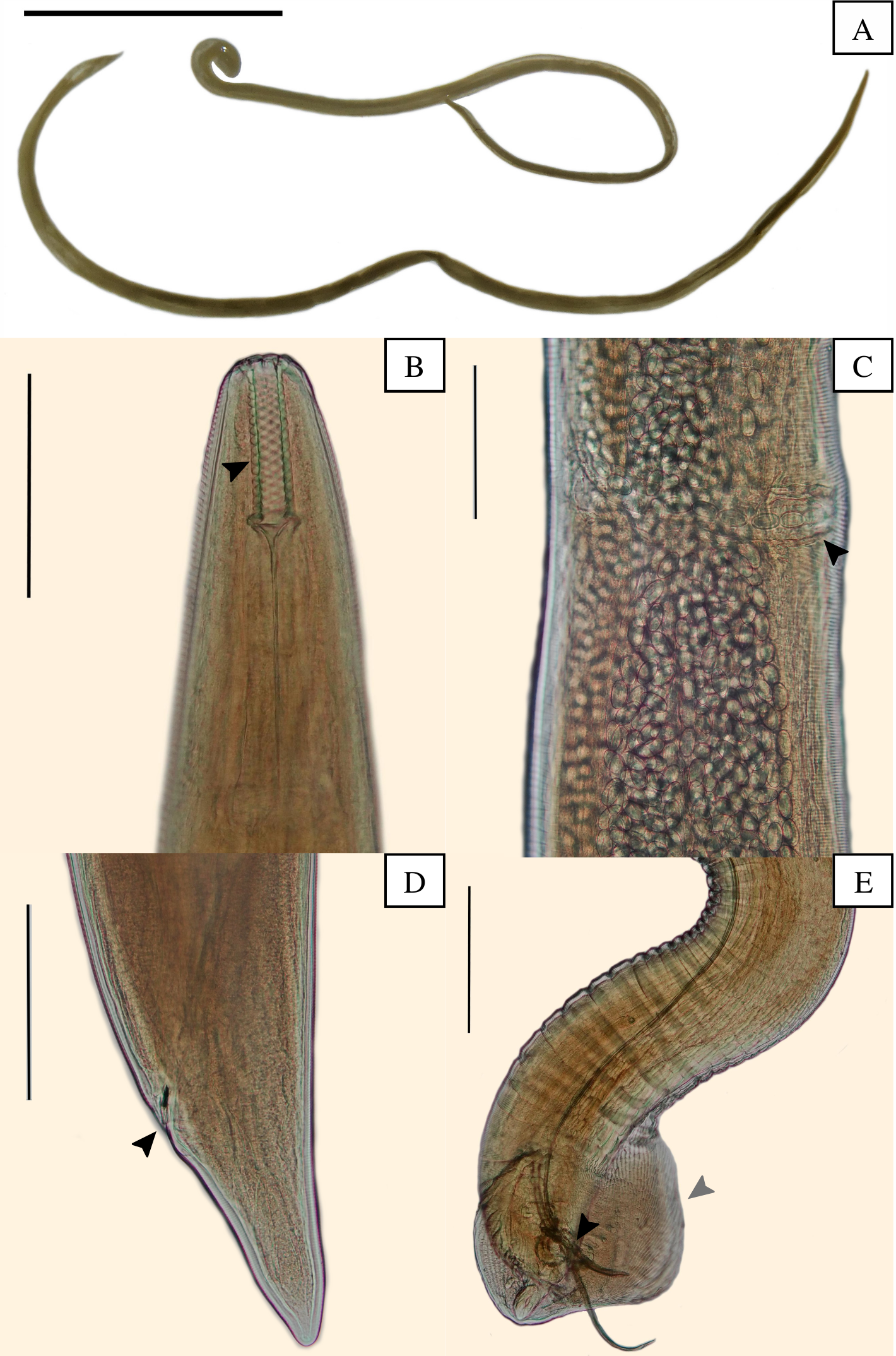

Supplement: Supplementary file 1 [file pathogens-12-00175-s001.zip › Figure S2.tiff]

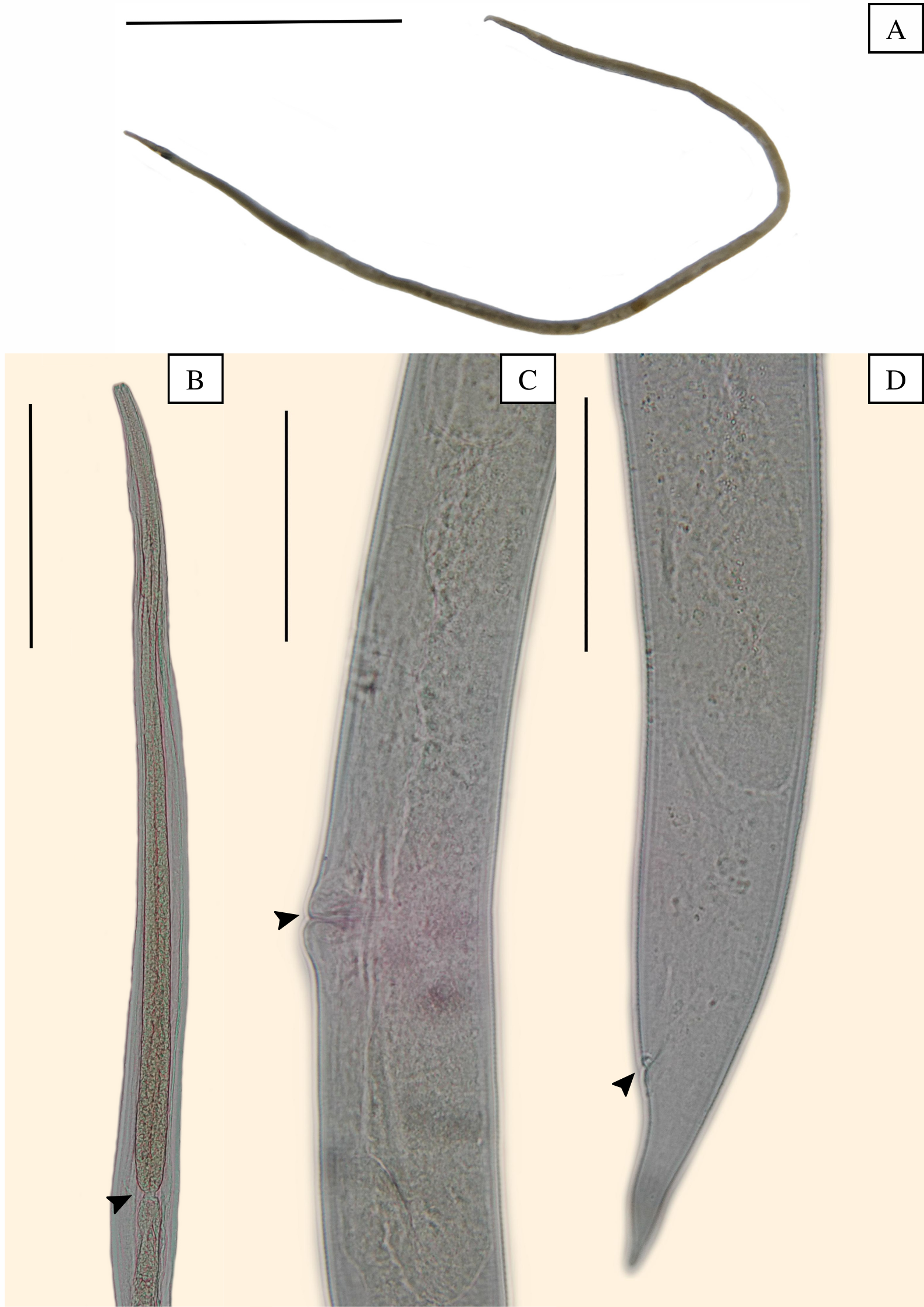

Supplement: Supplementary file 1 [file pathogens-12-00175-s001.zip › Figure S3.tiff]

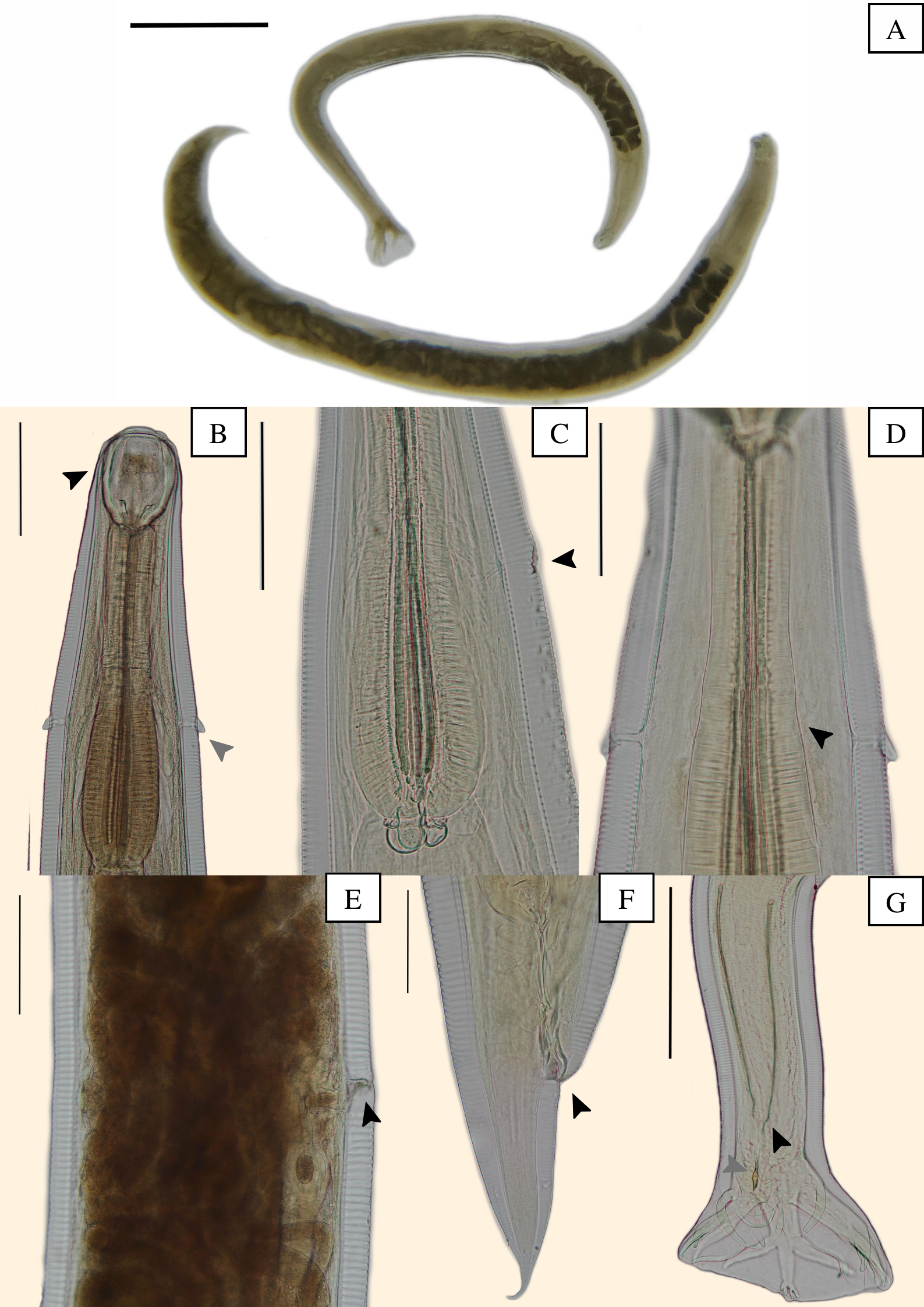

Supplement: Supplementary file 1 [file pathogens-12-00175-s001.zip › Figure S4.tiff]

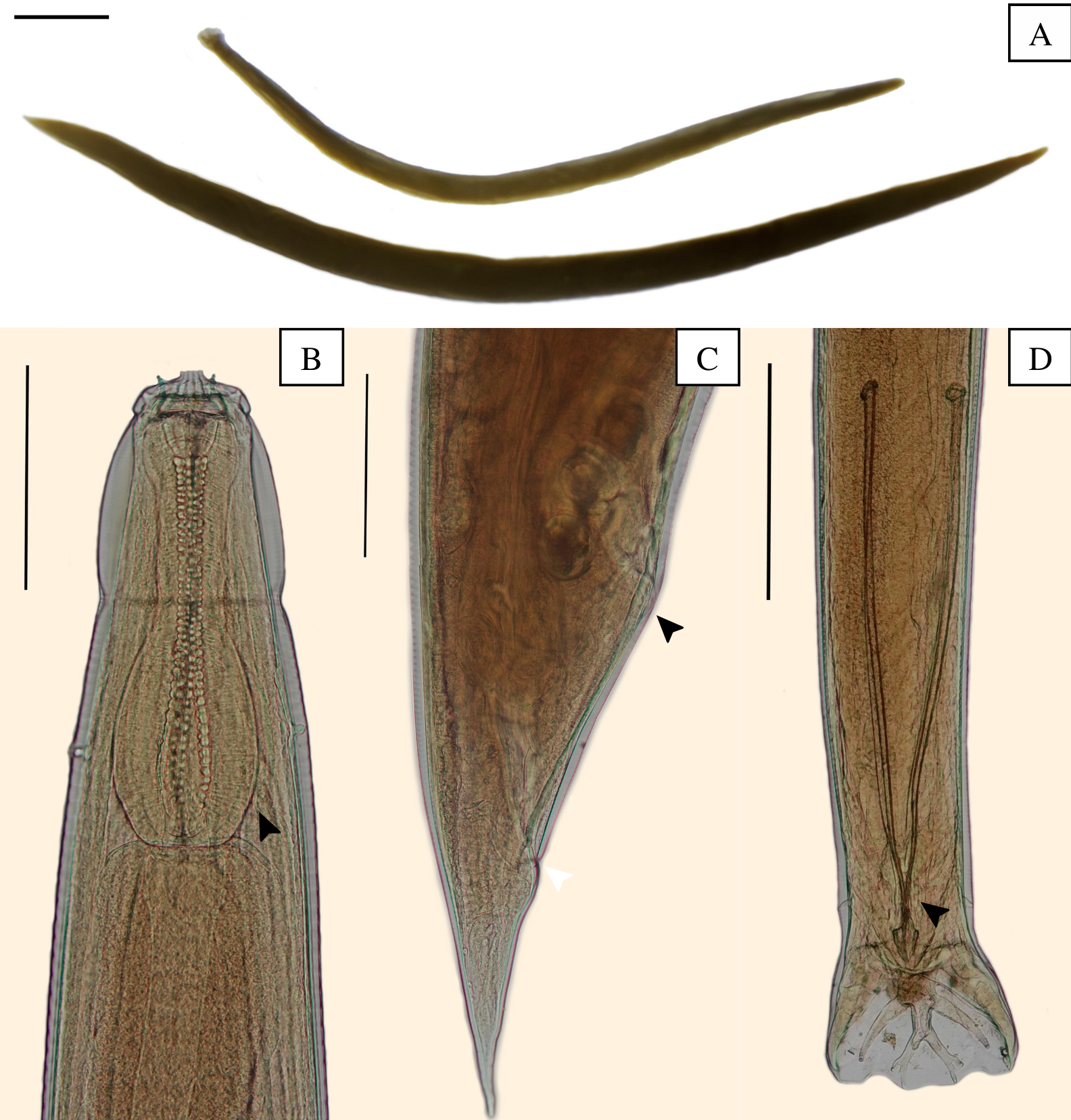

Supplement: Supplementary file 1 [file pathogens-12-00175-s001.zip › Figure S5.tiff]

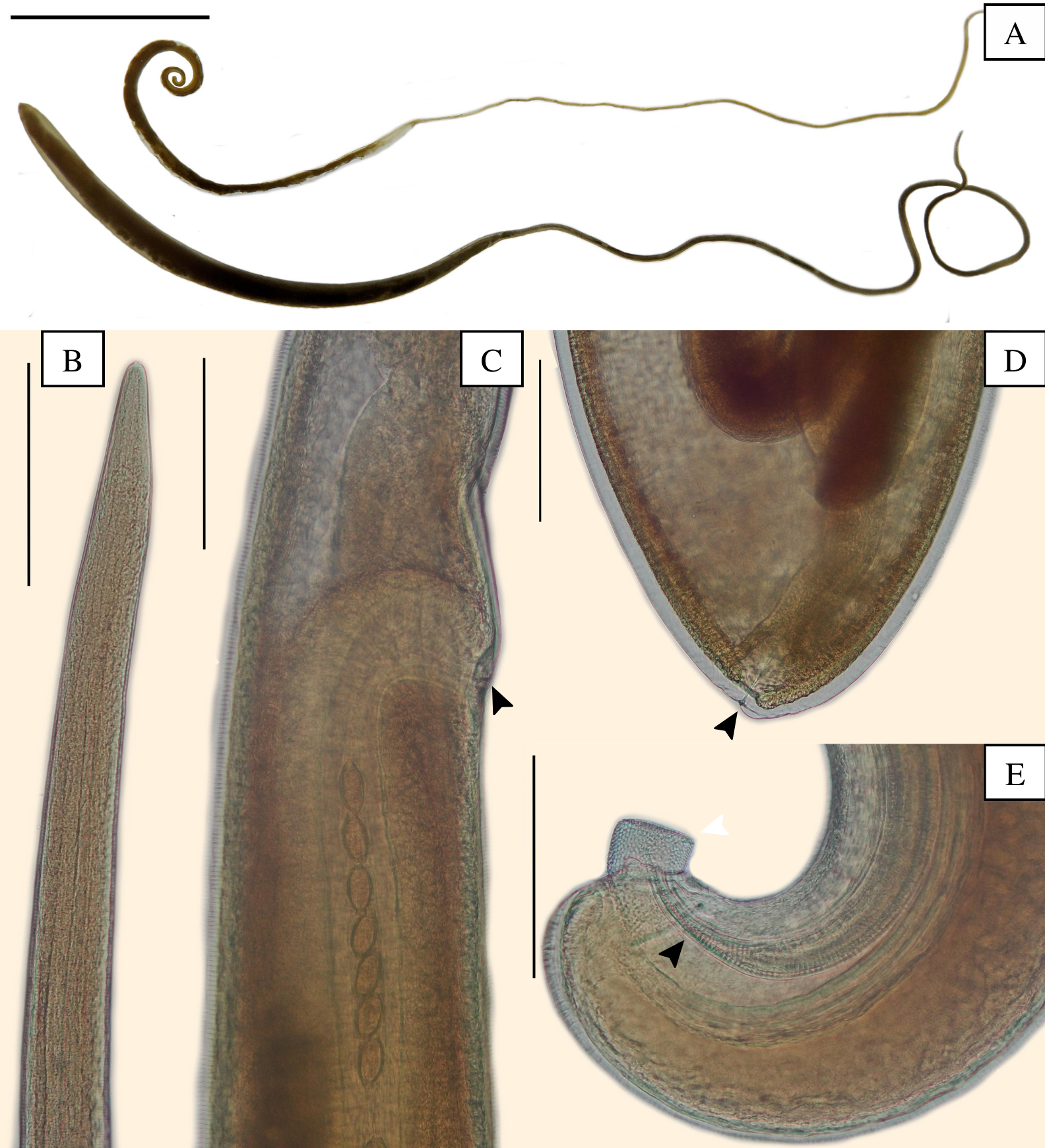

Supplement: Supplementary file 1 [file pathogens-12-00175-s001.zip › Figure S6.tiff]

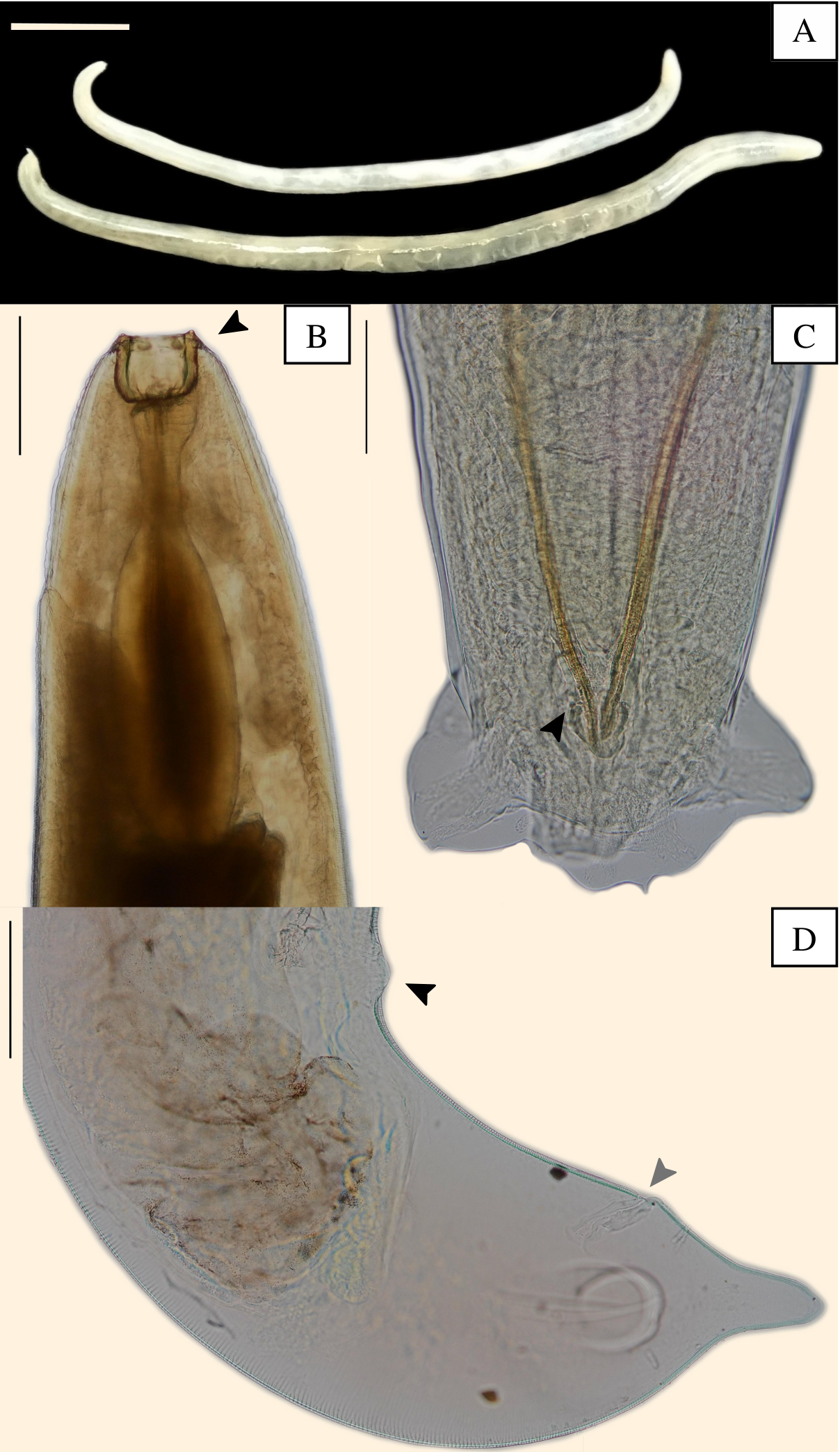

Supplement: Supplementary file 1 [file pathogens-12-00175-s001.zip › Figure S7.tiff]

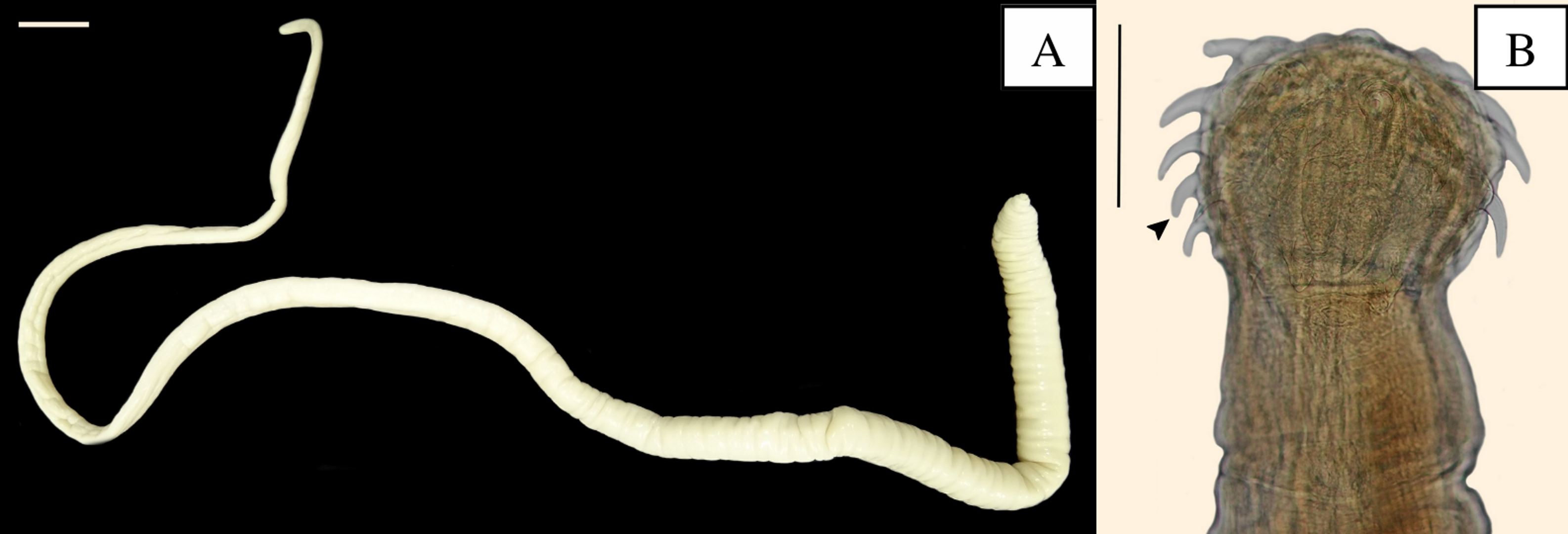

Supplement: Supplementary file 1 [file pathogens-12-00175-s001.zip › Figure S8.tiff]
